# Cancer awareness and socio-economic position: results from a population-based study in Denmark

**DOI:** 10.1186/1471-2407-14-581

**Published:** 2014-08-09

**Authors:** Line Hvidberg, Anette Fischer Pedersen, Christian Nielsen Wulff, Peter Vedsted

**Affiliations:** Research Centre for Cancer Diagnosis in Primary Care (CaP), Research Unit for General Practice, Department of Public Health, Aarhus University, Bartholins Allé 2, 8000 Aarhus C, Denmark; Section for General Medical Practice, Department of Public Health, Aarhus University, Bartholins Allé 2, 8000 Aarhus C, Denmark; Department of Oncology, Aarhus University Hospital, Noerrebrogade 44, 8000 Aarhus C, Denmark

**Keywords:** Denmark, Cancer, Awareness, Socio-economic position, Inequality

## Abstract

**Background:**

Differences in cancer awareness between individuals may explain variations in healthcare seeking behaviour and ultimately also variations in cancer survival. It is therefore important to examine cancer awareness and to investigate possible differences in cancer awareness among specific population subgroups. The aim of this study is to assess awareness of cancer symptoms, risk factors and perceived 5-year survival from bowel, breast, ovarian, and lung cancer in a Danish population sample and to analyse the association between these factors and socio-economic position indicators.

**Methods:**

A population-based telephone survey was carried out among 1,000 respondents aged 30–49 years and 2,000 respondents aged 50 years and older using the Awareness and Beliefs about Cancer measure. Information on socio-economic position was obtained by data linkage through Statistics Denmark. Prevalence ratios were used to determine the association between socio-economic position and cancer awareness.

**Results:**

A strong socio-economic gradient in cancer awareness was found. People with a low educational level and a low household income were more likely to have a lower awareness of cancer symptoms, cancer risk factors and the growing risk of cancer with age. Furthermore, men and people outside the labour force tended to be less aware of these factors than women and people within the labour force. However, women were more likely than men to lack awareness of the relationship between age and cancer risk. No clear associations were found between socio-economic position and lack of awareness of 5-year survival from bowel, breast, ovarian, and lung cancers.

**Conclusions:**

As cancer awareness has shown to be positively associated with cancer-related behaviour, e.g. healthcare seeking, consideration must be given to tackle inequalities in cancer awareness and to address this issue in future public health strategies, which should be targeted at and tailored to the intended recipient groups.

## Background

Large variations in cancer survival exist between countries across a range of cancer types. Survival rates are generally lower in Denmark than in comparable countries [1, 2]. Even within countries, survival rates vary much between patient groups with the same type of cancer, and for most cancers people with lower socio-economic position (SEP) have poorer outcomes than their socioeconomically more affluent counterparts [3, 4].

These variations between and within countries are undoubtedly multifactorial and complex, but a growing body of research suggests that differences in stage progression at the time of treatment initiation may explain some of this variation; thus, differences in the time that passes from the first symptom is experienced until diagnosis and treatment seem to play a crucial role [5, 6]. Recent years have seen a stronger focus on cancer awareness and its possible effect on the ‘patient interval’ (i.e. the time from the first symptom is experienced until healthcare is sought [7]) [8]. Across a range of different cancer types, both quantitative and qualitative studies have found an association between low awareness of cancer symptoms and risk factors, and a long patient interval [9, 10]. Studies have also indicated that the patient interval accounts for a substantial part of the time to diagnosis and treatment [11, 12]. Cancer awareness accordingly seems to be a potentially modifiable contributor to the variations seen in healthcare seeking and, ultimately, survival [8]. It is therefore important to assess cancer awareness among the general population and to investigate possible associations with different subgroups.

Few studies have explored cancer awareness in the general population, and they find that being a man, living alone, belonging to an ethnic minority group and having a low level of education are independently associated with a lower level of cancer awareness [13–15]. In these studies, all SEP indicators are based on self-reporting. Owing to the existence of a Civil Registration System (CRS) in Denmark with complete, updated information on all Danish citizens [16], a range of highly valid and complete SEP indicators can be linked to survey data at the individual level in this study.

The aim of the present study is to assess awareness of cancer symptoms, risk factors and perceived 5-year survival from bowel, breast, ovarian, and lung cancer in a Danish population sample and to analyse the association between these factors and several register-based SEP indicators.

## Methods

### Study population and data collection

Data on cancer awareness among the general population in Denmark were collected as part of the International Cancer Benchmarking Partnership (ICBP), Module 2 [17]. The survey consisted of a 20-minute computer-assisted telephone interview undertaken by trained native-language interviewers from the market research company Ipsos MORI using the Awareness and Beliefs about Cancer (ABC) measure [18].

**Figure 1:**
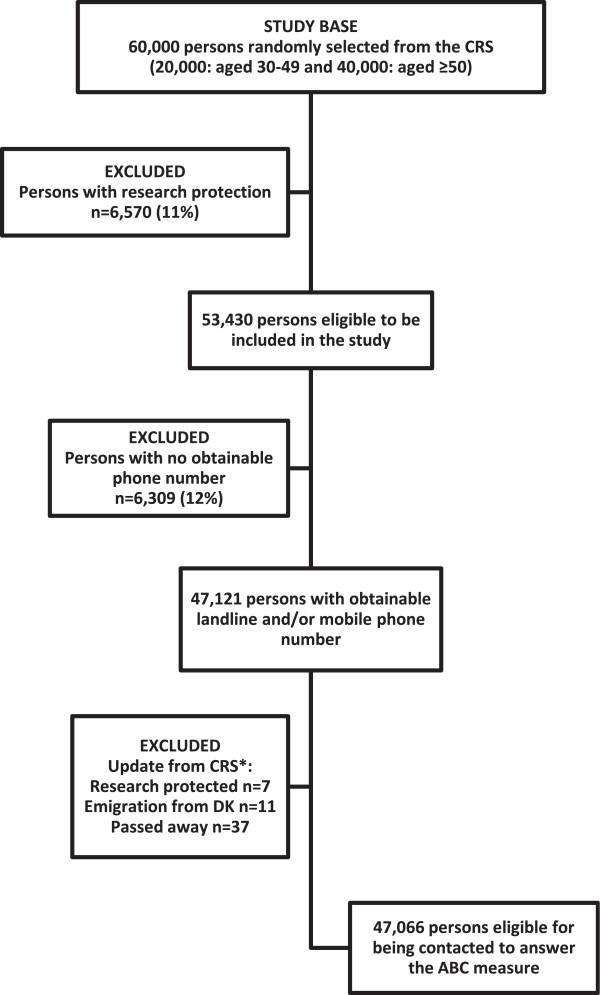
**Flow chart of survey population sampling.** *Before start of data collection, it was checked whether the persons 1) had a newly established research protection status, 2) had emigrated from Denmark or 3) had passed away.

In Denmark, a target study population of 1,000 respondents aged 30–49 years and 2,000 respondents aged 50 years and older was initially defined. Using the Danish CRS [16], a random study base was selected consisting of 20,000 persons aged 30–49 years and 40,000 persons aged 50 years and older. Among these 60,000 persons, a total of 6,570 persons (11.0%) were excluded because of research protection (i.e. publicly recorded rejection to be contacted for research purposes). For the remaining 53,430 persons, full name and complete address were obtained from the CRS and used by the Danish market research and consulting firm NN Markedsdata to obtain the phone number (landline and/or mobile) belonging to the identified person. Phone numbers could not be obtained for 6,309 (11.8%) persons, who were therefore excluded. Lastly, another 55 (0.1%) persons were excluded just before the data collection began, either because of a newly established research protection status, emigration from Denmark, or because the person had passed away. In total, 47,066 persons (78.4% of the study base) were thus eligible for being contacted to answer the ABC measure (Figure 1).

The Danish survey was conducted from 31 May to 4 July 2011, and each person was contacted on up to seven occasions at different weekdays and times. Interviews were not performed if the person was unable to speak or understand Danish.

### Survey measure

The ABC measure was applied; the development and the validation of the ABC measure is described elsewhere [18]. It explores awareness of cancer symptoms by using recognition as well as recall [19]; awareness of risk factors for cancer; awareness of growing risk of cancer with age; awareness of perceived 5-year survival from cancer; access to a doctor; anticipated healthcare seeking for cancer symptoms; anticipated barriers to healthcare seeking; beliefs about cancer in general; beliefs about cancer screening and actual screening behaviour. In addition, the measure explores smoking status, self-rated health and personal experience of cancer (own or close relatives, if any). The English version of the ABC measure was translated into Danish in accordance with the WHO guidelines for translation procedures, which included forward and backward translations [20].

### Dependent variables

Data reported here include awareness of cancer symptoms using the recognition method, awareness of risk factors for cancer, awareness of growing risk of cancer with age and awareness of 5-year survival from four different types of cancer.

#### Awareness of cancer symptoms

This awareness was measured by asking respondents whether they thought that a specific symptom could be a warning sign of cancer. In total, 11 different possible warning signs were stated in a rotated order with *yes*/*no* response options. These warning signs were unexplained lump or swelling; persistent, unexplained pain; unexplained bleeding; persistent cough or hoarseness; change in bowel or bladder habits; persistent difficulty in swallowing; change in the appearance of a mole; sore that does not heal; unexplained night sweats; unexplained weight loss; and unexplained tiredness. *Don’t know* was not indicated as a response category, but such a response was noted by the interviewer. A score of 1 point was given for a correct answer (*yes*), while an incorrect answer (*no*) was given 0 points. *Don’t know* was classified as an incorrect answer. The total score of cancer symptom awareness was computed (possible range: 0–11) and dichotomised into *low* and *high* awareness using the median split.

#### Awareness of risk factors for cancer

In a rotated order, respondents were asked whether they thought that a specific factor could increase their risk of getting cancer. Respondents could answer *strongly disagree*, *tend to disagree*, *tend to agree*, or *strongly agree* for 13 different risk factors: Smoking; exposure to passive smoking; drinking more than 1 unit of alcohol a day; eating less than 5 portions of fruit and vegetables a day; eating red or processed meat once a day or more; being obese; getting sunburnt more than once as a child; being over 70 years old; having a close relative with cancer; infection with human papillomavirus (HPV); not doing much physical activity; using a solarium; and exposure to ionising radiation from, for example, radioactive materials, x-rays, or radon. The answers *tend to agree* and *strongly agree* were given 1 point; and *strongly disagree*, *tend to disagree and don’t know* were given 0 points (possible range: 0–13). On the basis of the median split, awareness of risk factors for cancer was categorised into *low* and *high* awareness.

#### Awareness of growing risk of cancer with age

This element was assessed by asking the respondents the following question: “Over the next year, which of these groups of people, if any, do you think is most likely to be diagnosed with cancer?”. Four possible response categories were given: *30-year-olds*, *50-year-olds*, *70-year-olds,* or *people of any age are equally likely to be diagnosed with cancer*. Again, a response of *don’t know* was accepted, but was not mentioned by the interviewer. The answer *70-year-olds* was coded as correct, while all other answers were coded as *incorrect*.

#### Awareness of 5-year survival from four different types of cancer

Respondents were asked to estimate the 5-year survival rate from four different types of cancer: “Out of 10 people diagnosed with bowel/breast/ovarian/lung cancer, how many do you think would be alive five years later?” The interviewers recorded the stated number of people (0–10), and the following answers were coded as correct: Bowel (4–5), breast (8–9), ovarian (3–4) and lung (1–2) [21, 22]. To analyse the possible association between SEP and awareness of survival from the four different types of cancer, the data were dichotomised into *correct estimation* and *underestimation/overestimation*.

### Independent variables

Information on SEP indicators was obtained by data linkage to Statistics Denmark [23]. For each person in the study population, we obtained information on seven SEP indicators: gender (*female, male*); age (*30*–*49, 50–69* and *70+ years*); marital status (*married/cohabiting, living alone*); ethnicity (*ethnic Dane, immigrant/descendant*); level of education (*low*: ≤10 years, *middle*: >10 ≤ 15 years and *high*: >15 years) according to UNESCO’s International Standard Classification of Education (ISCED) groups [24]; occupation (*in the labour force*: employed and students, *outside the labour force*: unemployed, early retirement pensioner, disability retirement pensioner, personal or sick leave and *retired*: voluntarily retired person (special or old-age pensioner); and, lastly, OECD-modified disposable household income adjusted for number of adults and children in the household [25]. To level out yearly variation, this SEP indicator was calculated as an average for the preceding three years and categorised as low, middle and high income (*low*: ≤16,536 £/year, *middle*: >16,536 ≤ 33,095 £/year and *high*: >33,095 £/year) based on the 20%, 60% and 20% income distribution among the 60,000 persons in the study base. To examine the association between previous personal experiences with cancer and cancer awareness, we retrieved data from the Danish Cancer Registry on all registered cancer diagnoses (*yes*/*no*) for each person within the past 10 years [26]. For these register-based SEP indicators, missing data ranged from 0% for age, gender and information on cancer diagnosis to 3.9% for information about educational level for the study base of 60,000 persons. Data on close relatives with cancer (*yes*/*no*) and self-rated health (*very good*, *good*, *fair*, *poor* and *very poor* dichotomised into *good* and *fair/poor*) were obtained from the ABC survey.

### Statistical analysis

Analyses were carried out using Stata version 13.1. Statistical analyses were performed with unweighted data, because weighting may introduce additional bias. Prevalence ratios (PRs) with 95% confidence intervals (95% CIs) were used to determine the association between SEP indicators and awareness of cancer symptoms, risk factors for cancer, growing risk of cancer with age and 5-year survival for four different types of cancer. Unadjusted analyses were carried out with each of the independent variables, and an adjusted model was used to control for possible confounding. PRs were chosen over odds ratios (ORs) as ORs may overestimate the associations when there is a high prevalence of the dependent variables [27].

### Ethics and approval

The study was approved by the Danish Data Protection Agency (J. no. 2011-41-6237) and the Danish Health and Medicines Authority. In accordance with the Central Denmark Region Committees on Biomedical Research Ethics, the study needed no further approval (Report no. 128/2010).

## Results

### Response

To obtain inclusion of 1,000 respondents aged 30–49 years and 2,000 respondents aged 50 years or older, we approached a random sample of 11,297 persons. A response rate of 36.7% was achieved (Table 1); this was estimated as the number of completed interviews divided by the number of eligible persons made contact to. The SEP of the respondents and of the study base are shown in Table 2. A higher proportion of the respondents than of the entire study base of 60,000 persons were females, younger, married/cohabiting, ethnic Danes, had a high-level education, a high household income and were in the labour force. The differences were statistically significant at the 0.01 level.

**Table 1:** **Response rate**

Total number of persons approached	11,297
Number of ineligible persons	1,697^a^
Number of persons who could not be contacted after seven attempts	1,431
*Number of persons eligible and made contact to*	*8,169*
Number of persons who refused or did not complete the interview	5,169^b^
*Completed interviews*	*3,000*

^a^Incomplete/unobtainable number (n = 1,328); wrong number (n = 326); business/fax number (n = 8); number barred (n = 2); and unable to speak or understand Danish (n = 33).

^b^Refused to take part (before or after it was known whether or not it was the person eligible for study participation) (n = 4,736); stopped the interview (n = 154); the person eligible for study participation asked to be called back at a later date, but could not be contacted again (n = 141); the persons answering the phone did not want to speak to the interviewer (n = 92); another stated that the person eligible for the study was not available during data collection period (n = 31); and the person stated that he/she was not in the age group anyway (n = 15).

**Table 2:** **Socio-economic position (SEP) of the respondents (n = 3,000) and of the study base (n = 60,000)**

SEP indicators	Respondents n = 3,000		Study base n = 60,000		***P***value ^a^
	% (n)		% (n)		
***Gender***					<0.01
** Female**	55.3	(1,659)	51.5	(30,928)	
** Male**	44.7	(1,341)	48.5	(29,072)	
***Age group (years)***					<0.01
** 30-49**	33.3	(1,000)	33.3	(20,000)	
** 50-69**	50.3	(1,510)	46.2	(27,711)	
** ≥ 70**	16.3	(490)	20.5	(12,289)	
**Age, mean (SD)**	55.9	(13.3)	56.7	(15.1)	
***Marital status***					<0.01
** Married/cohabiting**	76.8	(2,303)	67.5	(40,449)	
** Living alone**	23.2	(695)	32.5	(19,464)	
***Ethnicity***					<0.01
** Ethnic Danes**	95.9	(2,876)	92.2	(55,215)	
** Immigrant/descendant**	4.1	(122)	7.8	(4,698)	
***Educational level***					<0.01
** High**	32.3	(954)	22.5	(12,988)	
** Middle**	46.2	(1,365)	47.1	(27,189)	
** Low**	21.5	(634)	30.4	(17,503)	
***Occupation***					<0.01
** In the labour force**	62.6	(1,844)	56.5	(33,027)	
** Outside the labour force**	8.1	(238)	11.2	(6,557)	
** Retired**	29.3	(864)	32.3	(18,844)	
***OECD-modified household income***					<0.01
** High**	25.2	(752)	20.0	(11,880)	
** Middle**	63.6	(1,902)	60.0	(35,641)	
** Low**	11.2	(335)	20.0	(11,880)	
***Cancer diagnosis within 10 years***					0.066
** Yes**	8.6	(258)	7.7	(4,636)	
** No**	91.4	(2,742)	92.3	(55,364)	
***Close relative(s) with cancer***					
** Yes**	78.1	(2,342)	-	-	
** No**	21.9	(656)	-	-	
***Self-rated health***					
** Good**	78.0	(2,334)	-	-	
** Fair/poor**	22.0	(659)	-	-	

Note: Numbers vary due to missing data.

^a^Chi-square test. One of the assumptions for this test is that observations are independent of each other. Therefore, we tested the difference between respondents and the study base without the respondents i.e. 57,000 persons.

### Awareness of cancer symptoms

The two most well-known symptoms of cancer were a change in the appearance of a mole and an unexplained lump or swelling. These two symptoms were recognised by 97.2% and 94.3% of the respondents, respectively. The lowest awareness of cancer symptoms was found for unexplained night sweats (15.6%) and a sore that does not heal (67.8%)*.*

The median number of cancer symptoms recognised by the respondents was nine out of 11. The associations between SEP and recognition of less than nine symptoms of cancer are presented in Table 3. In both the unadjusted and adjusted analyses, several of the SEP indicators were statistically significantly associated with awareness of cancer symptoms. Especially men, immigrant/descendants, people with low-level education, people outside the labour force, people with a low household income and people with no close relatives with cancer were more likely to recognise less than nine symptoms of cancer than women, ethnic Danes, people with a high-level education, people in the labour force, people with a high household income and people with close relatives with cancer, respectively.

**Table 3:** **Recognition of less than nine symptoms of cancer and associations with socio-economic position (SEP) indicators**

	Awareness of <9 symptoms of cancer	
SEP indicators	PR _unadj._ (95% CI)	PR* _adj._ (95% CI)
***Gender***		
** Female**	1.00	1.00
** Male**	**1.34 (1.24-1.46)**	**1.30 (1.20-1.41)**
***Age group (years)***		
** 30-49**	1.00	1.00
** 50-69**	0.92 (0.84-1.01)	**0.86 (0.78-0.94)**
** ≥ 70**	1.08 (0.96-1.21)	0.94 (0.83-1.06)
***Marital status***		
** Married/cohabiting**	1.00	1.00
** Living alone**	1.08 (0.98-1.18)	1.08 (0.98-1.18)
***Ethnicity***		
** Ethnic Danes**	1.00	1.00
** Immigrant/descendant**	**1.36 (1.16-1.60)**	**1.28 (1.08-1.50)**
***Educational level***		
** High**	1.00	1.00
** Middle**	**1.51 (1.36-1.69)**	**1.45 (1.30-1.62)**
** Low**	**1.58 (1.40-1.79)**	**1.57 (1.39-1.78)**
***Occupation***		
** In the labour force**	1.00	1.00
** Outside the labour force**	**1.21 (1.05-1.39)**	1.27 (1.11-1.46)**
** Retired**	1.08 (0.98-1.19)	1.13 (0.99-1.29)**
***OECD-modified household income***		
** High**	1.00	1.00
** Middle**	**1.22 (1.09-1.36)**	1.19 (1.07-1.33)**
** Low**	**1.32 (1.14-1.53)**	1.33 (1.15-1.54)**
***Cancer diagnosis within 10 years***		
** Yes**	1.00	1.00
** No**	1.02 (0.88-1.19)	1.07 (0.92-1.25)
***Close relative(s) with cancer***		
** Yes**	1.00	1.00
** No**	**1.22 (1.12-1.34)**	**1.17 (1.07-1.28)**
***Self-rated health***		
** Good**	1.00	1.00
** Fair/poor**	**1.10 (1.00-1.21)**	1.02 (0.93-1.13)

Note: Prevalence ratios (PR) with 95% confidence intervals (CI). Numbers in bold are significant results.

*Adjusted for gender, age, marital status, ethnicity, educational level, cancer diagnosis within the past 10 years, close relative(s) with cancer and self-rated health.

**Not adjusted for educational level due to intermediary associations between the variables.

Sensitivity analyses revealed similar social gradients in awareness of cancer symptoms based on recognition of both less than five and less than seven symptoms, but the PRs were generally higher in these analyses. For example, the PR of recognising less than five symptoms was 3.81 (95% CI 2.23-6.53) *(adjusted model; data not shown)* when people with a low-level education were compared with people with a high-level education. The corresponding PR was 1.57 (95% CI: 1.39-1.78) when the cut-off used was fewer than nine symptoms.

### Awareness of risk factors for cancer

The highest awareness of risk factors for cancer was found for smoking (96.5%) and using a sunbed (95.5%), while the lowest awareness was found for infection with HPV (23.6%) and intake of less than five daily portions of fruit and vegetables (41.0%).

The median number of risk factors recognised by respondents was nine out of 13. Certain characteristics were strongly associated with recognising less than nine risk factors for cancer; these include being a man, older, having a low-level education, or having a low household income (Table 4). Again, sensitivity analyses showed a stronger social gradient *(data not shown).*

**Table 4:** **Recognition of less than nine risk factors for cancer and associations with socio-economic position (SEP) indicators**

	Awareness of <9 risk factors for cancer	
SEP indicators	PR _unadj._ (95% CI)	PR* _adj._ (95% CI)
***Gender***		
** Female**	1.00	1.00
** Male**	**1.13 (1.05-1.21)**	**1.10 (1.02-1.18)**
***Age group (years)***		
** 30-49**	1.00	1.00
** 50-69**	**1.16 (1.07-1.27)**	**1.11 (1.02-1.21)**
** ≥ 70**	**1.36 (1.23-1.51)**	**1.23 (1.10-1.37)**
***Marital status***		
** Married/cohabiting**	1.00	1.00
** Living alone**	**1.12 (1.04-1.22)**	1.05 (0.96-1.14)
***Ethnicity***		
** Ethnic Danes**	1.00	1.00
** Immigrant/descendant**	1.14 (0.97-1.35)	1.09 (0.91-1.30)
***Educational level***		
** High**	1.00	1.00
** Middle**	**1.40 (1.27-1.54)**	**1.35 (1.22-1.48)**
** Low**	**1.53 (1.38-1.70)**	**1.40 (1.26-1.57)**
***Occupation***		
** In the labour force**	1.00	1.00
** Outside the labour force**	**1.25 (1.11-1.42)**	1.17 (1.02-1.33)**
** Retired**	**1.29 (1.20-1.40)**	1.13 (1.01-1.28)**
***OECD-modified household income***		
** High**	1.00	1.00
** Middle**	**1.19 (1.08-1.31)**	1.19 (1.07-1.33)**
** Low**	**1.38 (1.22-1.56)**	1.33 (1.15-1.54)**
***Cancer diagnosis within 10 years***		
** Yes**	1.00	1.00
** No**	1.02 (0.89-1.17)	1.06 (0.93-1.21)
***Close relative(s) with cancer***		
** Yes**	1.00	1.00
** No**	0.98 (0.89-1.07)	0.94 (0.86-1.03)
***Self-rated health***		
** Good**	1.00	1.00
** Fair/poor**	**1.15 (1.06-1.25)**	1.06 (0.98-1.16)

Note: Prevalence ratios (PR) with 95% confidence intervals (CI). Numbers in bold are significant results.

*Adjusted for gender, age, marital status, ethnicity, educational level, cancer diagnosis within the past 10 years, close relative(s) with cancer and self-rated health.

**Not adjusted for educational level due to intermediary associations between the variables.

### Awareness of growing risk of cancer with age

In total, 24% of the respondents were aware that 70-year-olds are more likely to be diagnosed with cancer than 30-year-olds, 50-year-olds and people of any age. However, the majority (42%) responded that people of any age are equally likely to be diagnosed with cancer or that 50-year-olds are more likely (30%)*.* Being a woman, having a low level of education, and a low income were associated with non-recognition of growing risk of cancer with age (Table 5).

**Table 5:** **Lack of awareness of growing risk of cancer with age and associations with socio-economic position (SEP) indicators**

	Lack of awareness of growing risk of cancer with age	
SEP indicators	PR _unadj._ (95% CI)	PR* _adj._ (95% CI)
***Gender***		
** Female**	1.00	1.00
** Male**	**0.91 (0.87-0.95)**	**0.90 (0.83-0.98)**
***Age group (years)***		
** 30-49**	1.00	1.00
** 50-69**	0.98 (0.94-1.02)	0.95 (0.86-1.04)
** ≥ 70**	1.00 (0.94-1.06)	0.92 (0.81-1.05)
***Marital status***		
** Married/cohabiting**	1.00	1.00
** Living alone**	**1.08 (1.03-1.13)**	1.05 (0.95-1.16)
***Ethnicity***		
** Ethnic Danes**	1.00	1.00
** Immigrant/descendant**	**1.10 (1.01-1.19)**	1.08 (0.88-1.34)
***Educational level***		
** High**	1.00	1.00
** Middle**	**1.30 (1.23-1.37)**	**1.31 (1.18-1.45)**
** Low**	**1.40 (1.32-1.48)**	**1.42 (1.26-1.60)**
***Occupation***		
** In the labour force**	1.00	1.00
** Outside the labour force**	**1.14 (1.07-1.21)**	1.06 (0.91-1.23)**
** Retired**	1.01 (0.97-1.06)	1.01 (0.88-1.16)**
***OECD-modified household income***		
** High**	1.00	1.00
** Middle**	**1.17 (1.11-1.24)**	1.15 (1.04-1.28)**
** Low**	**1.23 (1.15-1.32)**	1.19 (1.02-1.39)**
***Cancer diagnosis within 10 years***		
** Yes**	1.00	1.00
** No**	1.00 (0.93-1.07)	1.00 (0.86-1.16)
***Close relative(s) with cancer***		
** Yes**	1.00	1.00
** No**	0.98 (0.94-1.03)	0.98 (0.88-1.09)
***Self-rated health***		
** Good**	1.00	1.00
** Fair/poor**	**1.08 (1.03-1.13)**	1.03 (0.93-1.14)

Note: Prevalence ratios (PR) with 95% confidence intervals (CI). Numbers in bold are significant results.

*Adjusted for gender, age, marital status, ethnicity, educational level, cancer diagnosis within the past 10 years, close relative(s) with cancer and self-rated health.

**Not adjusted for educational level due to intermediary associations between the variables.

### Awareness of 5-year survival from four different types of cancer

**Figure 2:**
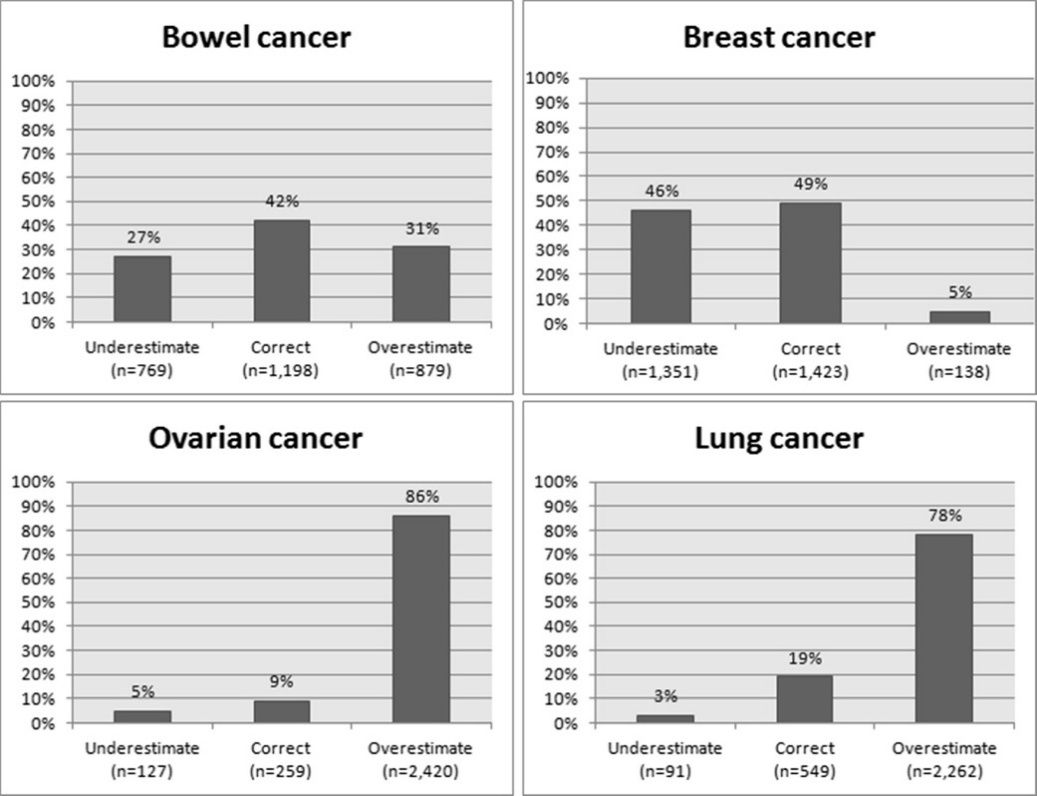
Proportion of respondents underestimating, correctly estimating and overestimating the 5-year survival from bowel, breast, ovarian, and lung cancer*. *Missing data for awareness of 5-year survival: bowel cancer: n = 154, breast cancer: n = 88, ovarian cancer: n = 194, lung cancer: n = 98, including response categories *don’t know* and *did not answer.*

The 5-year survival from bowel, breast, ovarian, and lung cancer was correctly identified by 42%, 49%, 9% and 19% of the respondents, respectively. For ovarian and lung cancer, a large majority (86 and 78%, respectively) of the respondents overestimated the 5-year survival, whereas almost half of the respondents underestimated survival from breast cancer. The distributions of the respondents’ estimations (underestimating, correctly estimating and overestimating) are shown in Figure 2.

Table 6 shows the associations between the SEP indicators and underestimation/overestimation of the 5-year cancer survival. People outside the labour force were more likely than people within the labour force to wrongly estimate the 5-year survival from breast cancer, and men were more likely to wrongly estimate the 5-year survival from lung and ovarian cancer than women. Furthermore, people with a low and middle income and people with no close relatives with cancer were less aware of the 5-year survival from bowel cancer than people with a high income and with close relatives with cancer.

**Table 6:** **Underestimation/overestimation of 5-year survival from bowel, breast, ovarian and lung cancer and associations with socio-economic position (SEP) indicators**

	Underestimation/overestimation of 5-year survival							
	Bowel cancer		Breast cancer		Ovarian cancer		Lung cancer	
SEP indicators	PR _unadj._ (95% CI)	PR* _adj._ (95% CI)	PR _unadj._ (95% CI)	PR* _adj._ (95% CI)	PR _unadj._ (95% CI)	PR* _adj._ (95% CI)	PR _unadj._ (95% CI)	PR* _adj._ (95% CI)
***Gender***								
** Female**	1.00	1.00	1.00	1.00	1.00	1.00	1.00	1.00
** Male**	1.02 (0.95-1.08)	1.03 (0.96-1.09)	0.98 (0.91-1.05)	0.97 (0.90-1.05)	**1.06 (1.03-1.08)**	**1.06 (1.03-1.08)**	**1.06 (1.02-1.09)**	**1.05 (1.01-1.09)**
***Age group (years)***								
** 30-49**	1.00	1.00	1.00	1.00	1.00	1.00	1.00	1.00
** 50-69**	0.96 (0.90-1.03)	0.96 (0.89-1.03)	1.03 (0.95-1.11)	1.02 (0.93-1.10)	0.99 (0.97-1.02)	0.99 (0.96-1.01)	1.05 (1.01-1.09)	**1.05 (1.00-1.09)**
** ≥ 70**	0.92 (0.84-1.02)	0.93 (0.83-1.03)	**1.15 (1.04-1.28)**	1.11 (0.99-1.24)	1.00 (0.97-1.04)	0.99 (0.96-1.03)	1.08 (1.02-1.14)	**1.06 (1.01-1.12)**
***Marital status***								
** Married/cohabitating**	1.00	1.00	1.00	1.00	1.00	1.00	1.00	1.00
** Living alone**	1.02 (0.95-1.10)	1.03 (0.96-1.11)	1.02 (0.94-1.11)	0.99 (0.91-1.08)	1.01 (0.99-1.04)	1.00 (0.97-1.03)	0.98 (0.94-1.02)	0.97 (0.93-1.02)
***Ethnicity***								
** Ethnic Danes**	1.00	1.00	1.00	1.00	1.00	1.00	1.00	1.00
** Immigrant/descendant**	0.97 (0.82-1.15)	0.98 (0.82-1.17)	1.11 (0.94-1.31)	1.08 (0.90-1.29)	0.95 (0.88-1.02)	0.93 (0.86-1.01)	1.03 (0.94-1.12)	1.02 (0.93-1.12)
***Educational level***								
** High**	1.00	1.00	1.00	1.00	1.00	1.00	1.00	1.00
** Middle**	1.03 (0.95-1.10)	1.02 (0.95-1.10)	1.02 (0.94-1.11)	1.02 (0.94-1.11)	**1.04 (1.01-1.07)**	**1.04 (1.01-1.06)**	1.01 (0.96-1.05)	1.00 (0.96-1.04)
** Low**	1.01 (0.92-1.11)	1.01 (0.92-1.11)	1.08 (0.98-1.19)	1.04 (0.94-1.16)	**1.04 (1.01-1.08)**	1.03 (1.00-1.07)	1.03 (0.99-1.09)	1.02 (0.97-1.07)
***Occupation***								
** In the labour force**	1.00	1.00	1.00	1.00	1.00	1.00	1.00	1.00
** Outside the labour force**	1.08 (0.96-1.21)	0.99 (0.88-1.12)**	**1.15 (1.01-1.31)**	1.15 (1.01-1.31)**	0.97 (0.92-1.02)	0.95 (0.90-1.00)**	1.00 (0.93-1.07)	1.00 (0.93-1.07)**
** Retired**	0.98 (0.91-1.06)	0.98 (0.88-1.09)**	**1.18 (1.09-1.27)**	1.12 (0.99-1.26)**	0.98 (0.95-1.01)	1.00 (0.97-1.02)**	1.04 (1.00-1.08)	0.98 (0.92-1.03)**
***OECD-modified household income***								
** High**	1.00	1.00	1.00	1.00	1.00	1.00	1.00	1.00
** Middle**	**1.12 (1.04-1.21)**	1.12 (1.04-1.21)**	1.00 (0.92-1.08)	1.00 (0.92-1.09)**	1.02 (0.99-1.05)	1.02 (0.93-1.12)**	1.00 (0.96-1.04)	1.00 (0.96-1.04)**
** Low**	**1.16 (1.03-1.30)**	1.18 (1.05-1.33)**	1.12 (0.99-1.26)	1.08 (0.95-1.23)**	1.02 (0.97-1.06)	1.02 (0.88-1.19)**	0.99 (0.92-1.05)	0.98 (0.91-1.04)**
***Cancer diagnosis within 10 years***								
** Yes**	1.00	1.00	1.00	1.00	1.00	1.00	1.00	1.00
** No**	1.04 (0.92-1.16)	1.00 (0.89-1.13)	0.90 (0.80-1.01)	0.94 (0.83-1.06)	0.99 (0.95-1.03)	0.99 (0.95-1.04)	0.99 (0.93-1.05)	0.99 (0.93-1.06)
***Close relative(s) with cancer***								
** Yes**	1.00	1.00	1.00	1.00	1.00	1.00	1.00	1.00
** No**	**0.91 (0.83-0.98)**	**0.91 (0.83-0.99)**	1.07 (0.99-1.16)	1.07 (0.98-1.16)	1.00 (0.97-1.03)	1.00 (0.97-1.02)	1.01 (0.97-1.06)	1.00 (0.96-1.04)
***Self-rated health***								
** Good**	1.00	1.00	1.00	1.00	1.00	1.00	1.00	1.00
** Fair/poor**	1.05 (0.97-1.13)	1.04 (0.97-1.13)	0.98 (0.90-1.07)	0.97 (0.89-1.06)	1.02 (0.99-1.05)	1.01 (0.98-1.03)	1.01 (0.97-1.05)	1.01 (0.97-1.05)

Note: Prevalence ratios (PR) with 95% confidence intervals (CI). Numbers in bold are significant results.

*Adjusted for gender, age, marital status, ethnicity, educational level, cancer diagnosis within the past 10 years, close relative(s) with cancer and self-rated health.

**Not adjusted for educational level due to intermediary associations between the variables.

## Discussion

A strong socio-economic gradient was found in cancer awareness; thus, people with a low educational level and a low household income were more likely to have a lower awareness of possible cancer symptoms, factors that can influence the risk of getting cancer, and the growing risk of cancer with age than people with a high-level education and people with a high household income. The sensitivity analyses showed that the associations between SEP and the respondents’ awareness of symptoms and risk factors were independent of the median cut-off; thus, the findings appear to be robust. We also saw a trend that men and people outside the labour force were less aware of these factors than were women and people in the labour force, respectively. However, women were more likely than men to lack awareness of the relation between age and cancer. No clear associations were found between SEP and lack of awareness of the 5-year survival from bowel, breast, ovarian, and lung cancer.

Our study supports findings from previous studies that people with a low SEP are generally more likely to be less aware of cancer than people with a high SEP [13, 15, 28, 29]. The findings also mirror the findings that cancer survival has a social gradient [3]. However, the mechanisms underlying the association between SEP and cancer awareness are not well understood. It has been suggested that, to some degree, the association may be related to health illiteracy and thus a lower capacity among people with lower SEP to obtain, process and understand health information [30].

It has also been rightly questioned whether a heightened awareness in itself may lead to the desired change in behaviour [31, 32]; knowledgeable people do not always make wise decisions [14, 33]. Recent research has also emphasised the role of other factors in the link between cancer awareness and cancer-related behaviour. Among others, it has been suggested that anticipated barriers to healthcare seeking and beliefs about cancer may mediate this link [33–35]. Although the role of cancer awareness as a determinant of behaviour should not be overemphasised, cancer awareness will often be an important step towards healthcare seeking and screening attendance [19, 36, 37].

The present study found that the two most commonly recognised symptoms of cancer were a change in the appearance of a mole and an unexplained lump or swelling and that smoking and sunbed use were the most well-known risk factors. On the other hand, unexplained night sweats and infection with HPV were the least recognised symptom and risk factor, respectively. These findings may reflect that Danish national campaigns have focused strongly on breast and skin cancers [38–40]. Thus, campaigns addressing cancer symptoms and risk factors may help the population evaluate these more accurately. Accurate evaluation of cancer symptoms and risk factors may reduce the patient interval [41, 42], increase screening uptake [43, 44] and encourage cancer risk-reducing actions [45, 46]. Our findings may also reflect the fact that a lump is a specific symptom, while unexplained night sweats, for example, are a less specific symptom that may be more readily associated with conditions such as menopause and infections than with cancer [47], and may therefore not immediately be considered a symptom of cancer. Likewise, in a comprehensive review by Macleod *et al.* [10], vague, ambiguous and more common symptoms were associated with a longer patient interval.

Cancer is primarily a disease of the elderly, and for most cancers the incidence rate increases with age [48]. However, the majority of the respondents tended to think that people of any age were equally likely to be diagnosed with cancer. This was a surprising finding; but as implied by others [37, 43], individuals may not conceptualise non-modifiable factors (such as age and gender) as risk factors, whereas modifiable factors (such as smoking and alcohol use) may be more easily seen as part of the conceptual framework for cancer risk among laypeople. Nevertheless, awareness about both modifiable and non-modifiable risk factors is important because awareness may facilitate healthcare seeking [28, 49].

Awareness of the 5-year survival from bowel and breast cancer was fairly high; however, only a small percentage of the respondents correctly identified the 5-year survival from ovarian and lung cancer. This may be due to inadequate communication about the chances of survival from these cancer types. However, the results for lung cancer may also be partly explained by end-aversion bias, i.e. the tendency to avoid the extremes of a scale.

### Strengths and limitations

A key strength of the present study was the use of the Danish CRS. All Danish residents are registered in the CRS which contains complete information on any Danish resident’s date of birth, gender, migration, etc. Owing to our use of the CRS, we were able to define a study base of 60,000 persons, a representative sample of the entire Danish population aged 30 years and older. Furthermore, the use of the CRS and the data linkage to a range of register-based SEP indicators provided us with precise and valid insight into variables that may be related to cancer awareness. Naturally, the SEP indicators capture correlated aspects. Still, since the correlation is not a hundred percent, each indicator contributes with unique information about the association with cancer awareness.

To analyse associations between SEP and cancer awareness of symptoms and risk factors, cancer awareness was categorised into low/high using the median split procedure. One of the shortcomings of this procedure is that the median is contingent upon the particular sample on which it is based [50, 51]. Thus, respondents categorised as having a low cancer awareness in this sample may be categorised as having a high cancer awareness in another sample. However, sensitivity analyses using both awareness of less than five and less than seven cancer symptoms and risk factors showed a similar, but intensified social gradient in cancer awareness.

A limitation of the study was the modest response rate. Only 36.7% of the persons whom we made contact to agreed to participate in the study. Unfortunately, response rates have been declining over the past decades and telephone surveys have been particularly affected by this decline [52]. However, by collecting data using a telephone interview, the respondent did not have the possibility to look for information elsewhere. This advantage could not have been achieved with paper-based or web-based surveys. The respondents completing the ABC measure were more often females, younger, married/cohabiting, had a high-level education and a high household income than people in the study base. As a consequence, selection bias may in some way affect the generalisability of the findings since women and persons with a high-level education and a high household income were generally more aware of cancer symptoms and risk factors than men and persons with a low educational level and a low household income. Consequently, the actual awareness level in the population is most probably lower than estimated here.

## Conclusion and implications

The results of this study indicate that people with a low educational level and a low household income are less aware of cancer than people with a high-level education and a high household income, respectively. Awareness about possible cancer symptoms, risk factors for developing cancer and survivability has shown to be positively associated with cancer-related behaviour, such as healthcare seeking and screening uptake. Thus, consideration must be given to tackle the current inequality in cancer awareness and to address this issue in future public health strategies. It is important that these strategies are targeted and tailored to the intended recipient groups. Otherwise, strategies may unintentionally increase social inequality in cancer awareness, as individuals with higher SEP often acquire and adapt to new health information at a faster rate than individuals with lower SEP [53].

In conclusion, decisions on healthcare seeking for potential cancer symptoms is a complicated process that is shaped by much more than simply awareness. Thus, the present study should be seen as part of a larger framework of research examining possible associations between SEP indicators and other factors that may influence cancer-related behaviour, such as beliefs about cancer and psychological and practical barriers to healthcare seeking.

## Abbreviations

ABC: Awareness and Beliefs about Cancer
CI: Confidence interval
CRS: Civil registration system
HPV: Human papillomavirus
ICBP: International Cancer Benchmarking Partnership
OR: Odds ratio
PR: Prevalence ratio
SEP: Socio-economic position.
